# Self-cleaning expanded polytetrafluoroethylene-based hybrid membrane for water filtration†

**DOI:** 10.1039/d2ra01026g

**Published:** 2022-05-04

**Authors:** Peng Liao, Lan You, Wen Jiang Zheng, Wei Zou, Jie Yan, Hu Yang, Fan Yang

**Affiliations:** Sichuan University of Science and Engineering Zigong 643000 PR China zhengwenjiang@suse.edu.cn; Organic Fluorine Material Key Laboratory of Sichuan Province, Zhonghao Chenguang Chemical Research Institute Zigong 643201 PR China

## Abstract

Membrane surface fouling is a key problem for water filtration. Compositing photocatalytic substances with a base membrane is a widely used strategy, but most of the membrane will be decomposed by photocatalysis. Herein, expanded polytetrafluoroethylene (ePTFE) with extremely stable chemical properties is grafted with polyacrylic acid (PAA) and then modified with titanium dioxide (TiO_2_) to realize a self-cleaning TiO_2_–PAA–ePTFE filtration membrane. It can recover its flux under UV irradiation after fouling. With 20 rounds of self-cleaning, the membrane microstructure still remains intact. Moreover, in addition to retaining bovine serum albumin, TiO_2_ particles on the membrane surface are capable of absorbing small organic pollutants and degrading them. Thus, this membrane is potentially used as an anti-fouling membrane for water filtration.

## Abstract 2

Herein, expanded polytetrafluoroethylene (ePTFE) with extremely stable chemical properties is grafted with titanium dioxide (TiO_2_) to realize a self-cleaning and UV resistance TiO_2_–PAA–ePTFE filtration membrane.

## Graphical abstract

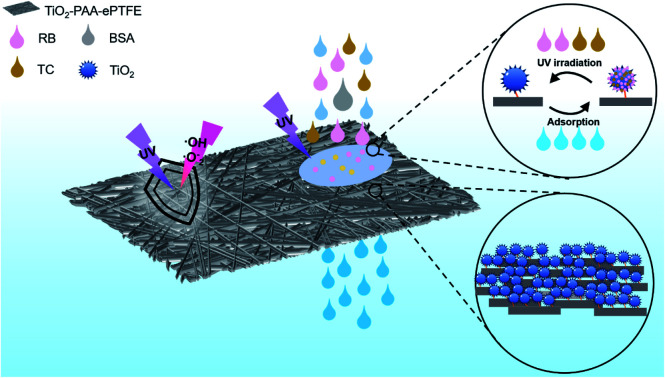

## Introduction

Membrane separation has been applied for water treatment worldwide because it is characterized as economic,^1^ energy-saving,^2^ easy to operate and high efficiency.^3,4^ Over the past decades, a series of membranes made of polyvinylidene fluoride,^5^ polyvinyl chloride,^6^ polysulfone,^7^ polystyrene,^8^ polyacrylonitrile^9^ and polypropylene have been developed to meet different filtration requirements. However, membrane fouling is still a key challenge in practice.^10^

Membrane fouling is generally attributed to organic foulants. Thus, a massive amount of work has focused on self-cleaning membranes through integrating photocatalysts. For example, Ayyaru *et al.*^11^ prepared PES–STiO_2_ membranes by a phase inversion method with the membrane flux of 502 to 802 L m^−2^ h^−1^; the recovery of water flux increased from 63% to 75.7 to 96.5%. Méricq J. *et al.*^12^ prepared PVDF–TiO_2_ film by a phase conversion method, which reduced the contact angle of the original film from 80° to 64°. After the membrane is polluted by BSA solution, the membrane performance can be completely restored by UV irradiation and water cleaning. Bojarska M. *et al.*^13^ prepared PP/plasma/ZnO film by a chemical bath deposition method, and studied the effect of the film on the degradation of methylene blue at different pH values. It was found that the best degradation effect was up to 90%. However, the photocatalytic function also brings about a key challenge for the membrane itself, because it can not only degrade the organic foulants but also attack the polymer structure of the membrane. Therefore, exploring a new catalysis resistant film material is urgent for self-cleaning membranes.^14^

Expanded polytetrafluoroethylene (ePTFE) is a kind of porous material with millions of pores per square centimeter that made by special biaxial stretching process.^15^ It inherits all the advantages of polytetrafluoroethylene with excellent stability and low surface energy and is a promising film material with catalysis resistant. However, its hydrophobic surface restricting the application in water treatment fields because the pores are not permeable to water. Several surface modification methods have been developed to address this problem, such as sodium-naphthalene treatment^16^ high energy ray activation,^17^ low temperature plasma-treatment.^18,19^ Among these methods, the last one is the most promising strategy for the modification of water treatment ePTFE membranes because it has scarcely no damage to the microstructure of the membrane.

In this work, the photocatalysts TiO_2_ particles were immobilized on ePTFE film through a facile two-step method, *i.e.*, surface grafting of polyacrylic acid (AA) and hydrolysis of tetrabutyl titanate. The resultant membrane can degrade the fouling organics and maintain the high flux for tens cycles of filtration under UV irradiation. And the microstructure of the film still remained intact. Moreover, the large specific surface area of the TiO_2_ particles enable to absorb organics that smaller than membrane pore size and degrading them.

## Experimental

### Materials

The ePTFE film is from China Chenguang Research Institute. Acrylic acid (AA, 99%), sodium dodecyl benzene sulfonate (SDBS, 98%), tetrabutyl titanate (TTB, 98%), absolute ethanol and acetone were purchased from Sinopharm Chemical Reagent Co., Ltd. Ultrapure water was applied in all experiment. All chemicals were analytical grade and used directly without any purification.

### Membrane modification

The ePTFE film was cut into a circle with a diameter of approximately 5.0 cm and rinsed with ethanol before use. Firstly, the ePTFE film was treated by Ar-plasma at a power of 100 W for 4 min to form the radicals on the surface. The ePTFE film was subsequently exposed to air for 120 min and used in subsequent surface modification experiments.

Plasma treated ePTFE film was first stuck inside a glass mold with size of 0.2 × 10 × 10 cm (thickness × width × height). The 20 wt% AA solution with sodium dodecyl benzenesulfonate (1.6 × 10^−3^ mol L^−1^) was then transferred to a glass mold, and nitrogen was applied in the solution of the glass mold for 5 min to remove dissolved oxygen. The glass mold was heated at 60 °C for 180 min.^20^ After modification experiment, the modified PTFE membranes were rinsed many times with ultra-pure water and dry it.

Modified PTFE film was put into a mixed solution of ethylene glycol (10 ml) and tetrabutyl titanate (0.1 ml). This solution was stirred for 8 h at a vacuum glove box. Subsequently, solution of ethanol (0.25 mL) and 98% H_2_SO_4_ (0.45 mL) were added to the above solution. Finally, 20 mL acetone was added and this mixture was transferred to a Teflon-lined autoclave and maintained at 180 °C for 6 h. After the experiment, the ePTFE film was washed repeatedly with isopropanol and ammonia solution.

### Membrane characterization

ATR-FTIR (Thermo Fisher Nicolet iS10, USA) was used to verify the modification of the membranes in the range of 4000–400 cm^−1^. X-ray photoelectron spectroscopy (XPS, Bruker D8 advance, Germany) analysis was carried out to analyze the elemental composition of the membranes. The morphologies and microstructures (SEM, Thermo Scientific Apreo 2C, USA) of membranes are characterized by emission scanning electron microscopy. Energy dispersive spectrometer (EDS, OXFORD ULTIM Max65, Britain) was used to analyze the distribution of various elements on the film. Atomic force microscope (AFM, Dimension ICON, USA) was used to measure the film surface roughness. Automatic specific surface area and pore size analyzer (American micrometrics ASAP 2460) was used to measure the specific surface area of titanium dioxide. High performance automatic mercury porosimeter (micrometrics 9600, USA) was used to measure membrane pore size. Precision electronic universal material testing machine (Shimadzu AGX-V, JP) was used to analyze the mechanical properties of the film. Synchronous thermal analyzer (Mettler Toledo TGA/DSC 3^+^, CH) was used to analyze the thermal properties of the films.

### Contact angle and surface energy test

The water contact angle of each membrane was measured by a contact angle goniometer (optical angle meter and interface tensiometer, USA). For each measurement, 2 μL drops were formed using ultra-pure water and the subsequently reported contact angles were taken as an average of at least three measurements. The Owens–Wendt–Rabel–Kaelble solid surface free energy estimation method in the test angle measuring instrument is selected to calculate the surface energy of three different membranes, in which ultrapure water and diiodomethane are used as the test liquid.

### Membrane flux test

The membrane flux of pure water was measured by dead end filtration experiment. The membrane in circle shape with a diameter of 4.6 cm was put it into a 50 ml ultrafiltration cup, and measured the membrane flux at 0.1 MPa after 30 minutes of pre operation at 0.15 MPa. The membrane flux is calculated according to eqn (1).1
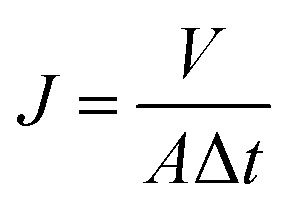
where *V* (L) is the permeate volume, *A* (m^2^) is the membrane filtration area, and Δ*t* (h) is the permeate time.

### Study on leaching and membrane stability

#### Leaching study

The prepared films were immersed in 10 ml ultra-pure water under the irradiation of LED ultraviolet lamp with the distance of 50 cm away from the film sample, in which the wavelength of ultraviolet lamp was 365 nm and the power was 60 W. Then the films were taken out at different times (up to 120 hours) and placed in the photocatalytic device. 50 ml ultrapure water was circularly filtered under the irradiation of LED ultraviolet lamp to determine the titanium dioxide filtered from the membrane during the filtration process. The filtration pressure was 0.1 MPa. 1 ml of filtrate at different times (up to 8 hours) was taken out and digested with hydrofluoric acid for titanium measurement using ICP-OES (PerkinElmer optima 8000 US).

#### Membrane stability experiment

The original ePTFE membranes were exposed to ultraviolet lamp for different times (up to 120 h) and adopted to membrane flux test and infrared test to investigate the anti-UV stability of the ePTFE base film. The mechanical stability was studied by mechanical test of the TiO_2_–PAA–ePTFE film after 20 cycles of filtration under UV condition, and the film without UV irradiation was also tested as control.

### Photocatalytic degradation of organic wastewater

Rhodamine B (RB), tetracycline hydrochloride (TC) and bovine serum protein (BSA) were used as representatives of organic wastewater to evaluate the photodegradation ability of TiO_2_–PAA–PTFE. Fix the membrane on the bottom of the quartz beaker with an iron ring, add RB (10 ml, 5 ppm)/TC (10 ml, 15 ppm)/BSA (15 ml, 10 ppm), and then place the quartz beaker in the photocatalytic instrument. Keep it in the dark for 30 min to make organic wastewater and membrane reach adsorption equilibrium, then turn on the photocatalytic instrument, take out 2 ml every 30 min and detect it with UV-vis spectrophotometer, and take back to the quartz beaker after testing. The protein content of bovine serum was measured by Coomassie brilliant blue method.^21^

### Synergistic effect of catalysis and adsorption and cyclic test

Rhodamine B (RB) and tetracycline hydrochloride (TC) were used as small molecule organic wastewater to test the synergistic effect of catalysis and adsorption of TiO_2_–PAA–ePTFE membrane. The instrument is composed of 50 ml ultrafiltration cup and UV-LED lamp band, which is circularly filtered with Rb (3 ppm, 20 ml)/TC (15 ppm, 20 ml). Under 0.01 MPa, 20 °C and UV-LED lamp, 2 ml filtrate is taken every 30 minutes for detection and put back. Cycle test for a total of 2 hours.

The circulation test is the same as the above operation. After each test, the membrane needs to be cleaned with ultrapure water and ethanol, and then repeat the next test. The length of each experiment was 3 hours, and a total of 5 experiments were carried out.

### Synergistic effect of catalysis and filtration and cyclic test

The photocatalytic filtration device is composed of a 50 ml ultrafiltration cup and a UV lamp with power of 60 W and wavelength of 365 nm. Bovine serum albumin (BSA) was used as a macromolecular organic wastewater to test the synergistic effect of catalysis and filtration of the TiO_2_–PAA–ePTFE membrane. Continuously pour 1 g L^−1^ BSA into the ultrafiltration cup and filter at 20 °C, 0.1 MPa until the membrane flux drops below 20%. Stop the filtration. Remove the film and operate: (1) After 25 minutes of LED UV irradiation, the membrane was immersed in ethanol for ultrasonic cleaning for 1 minute, and finally cleaned with ultrapure water; (2) the membrane was cleaned with ultrapure water, then immersed in ethanol for ultrasonic cleaning for 1 minute, and finally cleaned with ultrapure water. Record the time every 50 ml of filtrate collected during filtration. After each cycle test, perform operation (1) and then start the next cycle test.

## Results and discussion

**Scheme 1:**
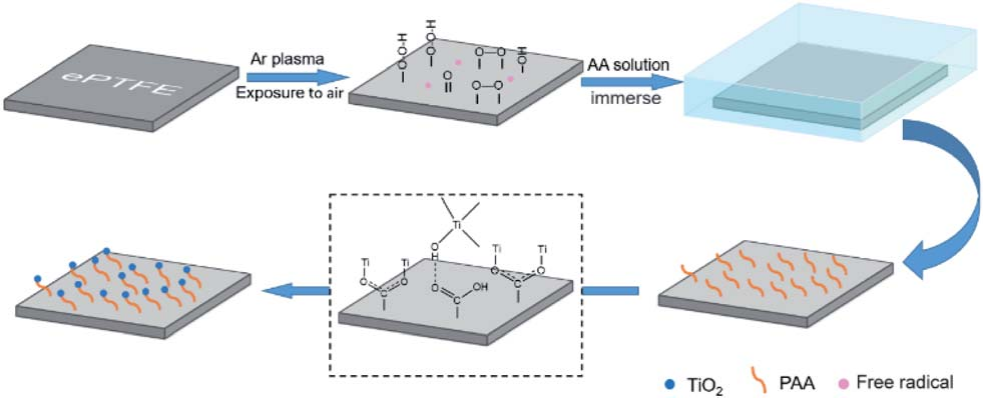
Schematic diagram of TiO_2_–PAA–ePTFE film preparation process.

ePTFE film has millions of pores per square centimeter,^15^ which is an ideal filter material. However, it is difficult to apply to the field of water filtration because of its super-hydrophobic nature.^22^ Herein, the ePTFE film is composited with hydrophilic titanium dioxide (TiO_2_) particles to improve its water permeability. As shown in Scheme 1, ePTFE film was modified with TiO_2_ particles through a two-step method. In step 1, polyacrylic acid (PAA) was grafted onto ePTFE film through low-temperature plasma technology. The Ar plasma-pretreated ePTFE films were exposed to air to form peroxide and hydroperoxide groups. Subsequently, these groups were decomposed into free radicals by heat and initiated the polymerization of acrylic acid (AA) monomers.^23^ In step 2, TiO_2_ particles were grown *in situ* on ePTFE film through solvothermal method with the advantage of the grafted PAA chains. In this step, the hydrolysis of tetrabutyl titanate (TiO(OH)_2_) was connected with PAA by ionic coordination and hydrogen bonding, and then grown into solid particles.^24,25^ As a result, the ePTFE film loaded with TiO_2_ was prepared and is abbreviated as TiO_2_–PAA–ePTFE in the following.

**Fig. 1:**
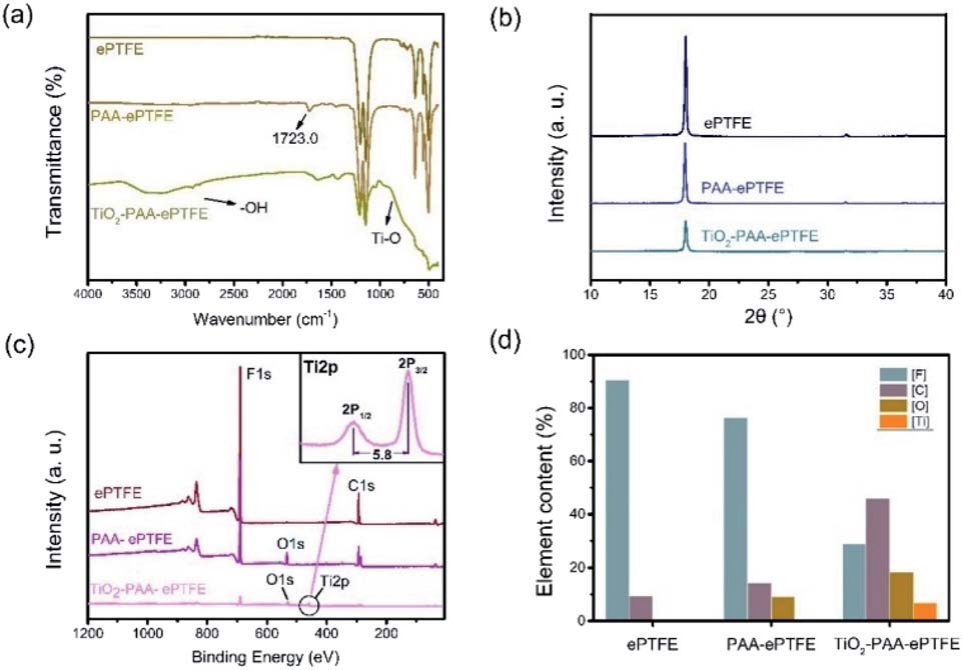
(a) FTIR spectra, (b) XRD spectra, (c) XPS spectra and high resolution Ti2p XPS spectra of TiO_2_–PAA–ePTFE film; (d) atomic percentage values of different PTFE film.

To verify the whole preparation process, the surface properties of the films in each preparing step were characterized. As Fig. 1a shows, infrared absorption at 1714 cm^−1^ appears in the spectrum of ePTFE film grafted with PAA (PAA–ePTFE), which is related to the stretching vibration of carbonyl groups, indicating that PAA chains have been successfully grafted onto ePTFE surface.^26,27^ After compositing with TiO_2_, the vibration and stretching of Ti–O bond appeared at 800 to 500 cm^−1^ in the spectrum of TiO_2_–PAA–ePTFE.^25,28^ In addition, a wide peak around 3300 cm^−1^ caused by Ti–OH interaction and surface absorbed water has also been observed. XRD was used to analysed the crystal structure of ePTFE films. Just as seen in Fig. 1b, the ePTFE characteristic peaks at 2*θ* = 17.99° were (1, 0, 0) crystal plan. The characteristic peak intensities of the ePTFE, PAA–ePTFE, and TiO_2_–PAA–ePTFE films decreased sequentially, which resulted from the destruction of the crystal forms on the surface of the films after grafting of PAA and TiO_2_. The chemical composition of ePTFE films surface was determined *via* XPS analysis. Fig. 1c shows that the original ePTFE film is mainly consisted of C and F elements which only has C1s and F1s peaks. After grafting PAA, O1s (534 eV) peak appeared, indicating that the carboxyl groups have been modified to the surface of ePTFE membrane. In the high-resolution Ti2p XPS spectra of TiO_2_–PAA–ePTFE films (Fig. 1c), double peaks appeared at Ti2p_1/2_ (464.4 eV) and Ti2p_3/2_ (458.6 eV), which accords with the Ti XPS in TiO_2_ reported in literature.^29^ In particular, the F1s peak intensity decreases sharply due to the increasing of O1s and Ti2p, indicating that TiO_2_ has been successfully grafted on the surface of ePTFE film, which agreed with the results of FT-IR results. Further, the atomic percentage values of all the films are shown in Fig. 1d. It can be seen that F atom decreases from 90.5% to 28.8%, O atom rises from 0 to 18.3%, and Ti atom accounts for 6.9% of the film surface.

**Fig. 2:**
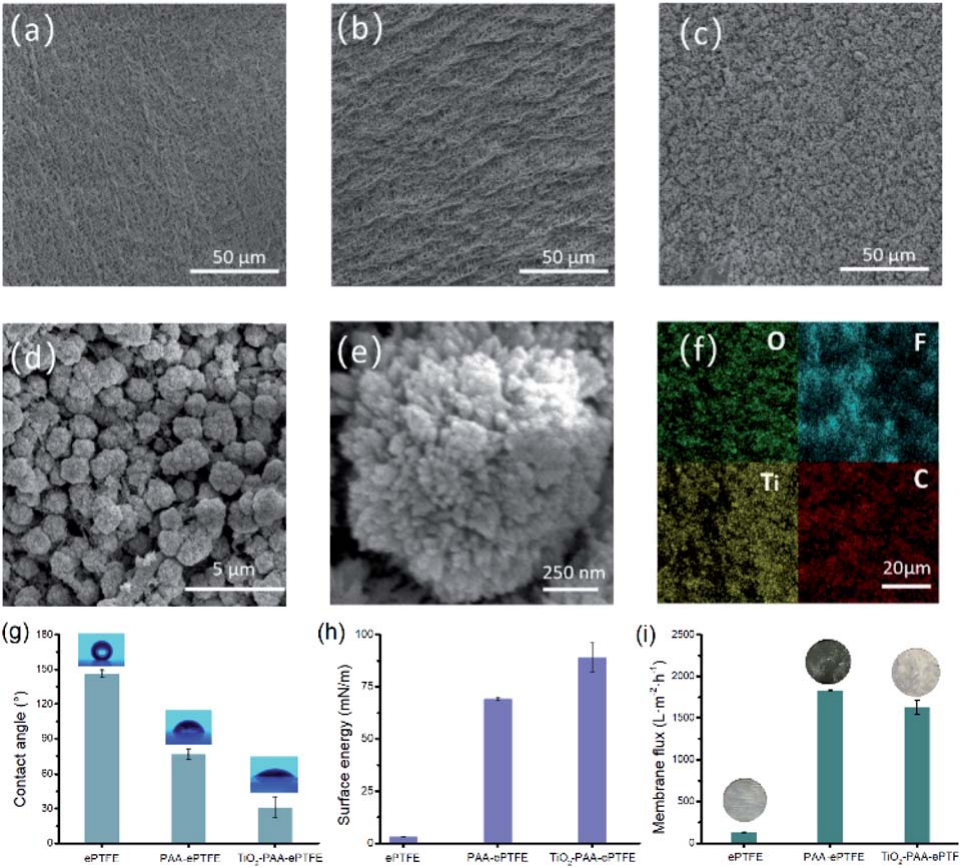
SEM images of different membrane surfaces. (a) Untreated ePTFE (×1k), (b) PAA–ePTFE (×1k), (c) TiO_2_–PAA–ePTFE (×1k), (d) TiO_2_–PAA–ePTFE (×20k), (e) TiO_2_–PAA–ePTFE (×50k); (f) EDS elemental maps measurements of the TiO_2_–PAA–ePTFE membrane; (g) water contact angles; (h) surface energy and (i) membrane flux of different membranes.

The surface morphology of modified ePTFE films was imaged using SEM. Fig. 2a shows that the ePTFE film has a microporous structure composed of a large number of interconnected fibers. After grafting with PAA, the fiber structure of ePTFE stays integrity and bulges appeared on its surface (Fig. 2b). The PAA–ePTFE has significantly increased roughness over the virgin ePTFE film (Fig. 2a). As shown in Fig. 2c, a large amount of hedgehog shaped TiO_2_ particles are observed on the surface of ePTFE, and covering the fibrous structure. The EDS scan images of the TiO_2_–PAA–PTFE films are shown in Fig. 2f. The results show that the Ti and O elements are uniformly distributed on the surface of the TiO_2_–PAA–ePTFE films. This indicates that TiO_2_ particles have been successfully prepared on ePTFE film. Fig. 2d shows a clear view of the TiO_2_–PAA–ePTFE film. The fibrous network is still visible and well combined with TiO_2_ particles. This is favorable for the water permeability of the film. As Fig. 2g shows, the original film has the characteristics of super hydrophobicity and ultra-low surface energy with water contact angle of ∼140°. With the modification of PAA and TiO_2_, the contact angles are obviously decrease to 76 ± 6° and 31 ± 8°, respectively, and the surface energy increased significantly from 3.2 mN m^−1^ to 89.1 mN m^−1^. In addition, the hedgehog shaped TiO_2_ shows many radial shaped needle-like antennae on the surface of the crystal nucleus (Fig. 2e), with increased adsorption capacity (specific surface area: 234.5 m^2^ g^−1^).^30,31^

Fig. 2i shows the membrane flux of water for films. In respected to the original ePTFE film, the membrane flux of the PAA–ePTFE and TiO_2_–PAA–ePTFE increased by nearly two orders of magnitude, which are 1835 ± 10 L m^−2^ h^−1^ and 1627 ± 84 L m^−2^ h^−1^, respectively. It can also be seen intuitively from the insert photos that the ePTFE film is completely opaque in water, and it becomes transparent after grafting with PAA. That is, water can easily penetrate through the modified PAA–ePTFE film. While, the opacity of the water-permeable TiO_2_–PAA–ePTFE is caused by a layer of white TiO_2_ particles formed on its surface. Compared with the original ePTFE film, the modified ePTFE shows a larger tensile strength (Fig. S1a†) and retains a nearly constant thermal stability (Fig. S1b†), which will be beneficial to filtration under high pressure (Fig. S1†).

**Fig. 3:**
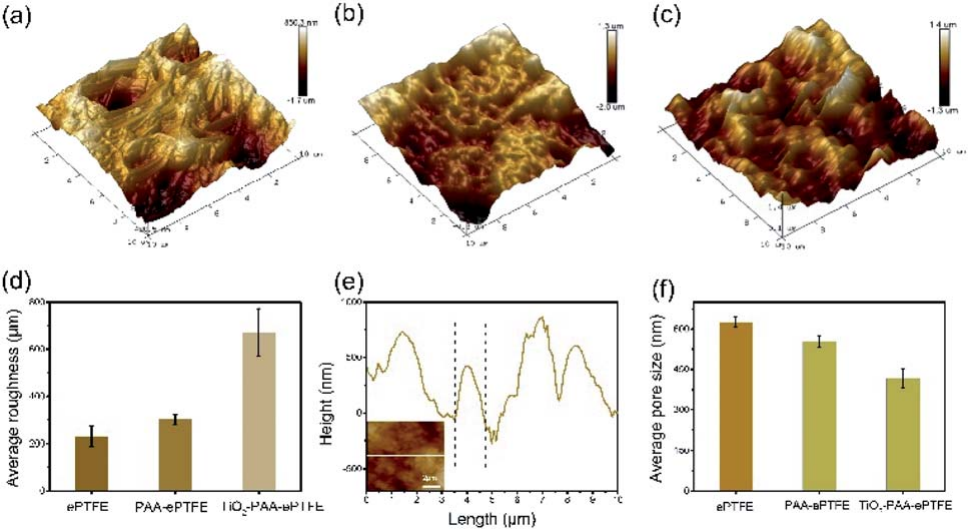
AFM of different membranes: (a) untreated ePTFE, (b) PAA–ePTFE, (c) TiO_2_–PAA–ePTFE; (d) roughness of different membranes; (e) AFM height image of TiO_2_–PAA–ePTFE; (f) average pore diameter of different membranes.

The surface topographies and roughness of the ePTFE films were observed with AFM (Fig. 3). It can be clearly seen that the AFM image of virgin ePTFE film has a microporous structure (Fig. 3a). After grafting with PAA (Fig. 3b), the fibrous structure disappeared and the roughness become much bigger. Then as loaded with TiO_2_ particles, the TiO_2_–PAA–ePTFE shows the roughest surface over the previous two groups (Fig. 3c and e) and the pores become smallest (Fig. 3f). From the line scanning path (Fig. 3d), it can be seen that the width of the bulge on the TiO_2_–PAA–ePTFE surface is about 1 μm, which is consistent with the particle size of TiO_2_ observed in SEM.

**Fig. 4:**
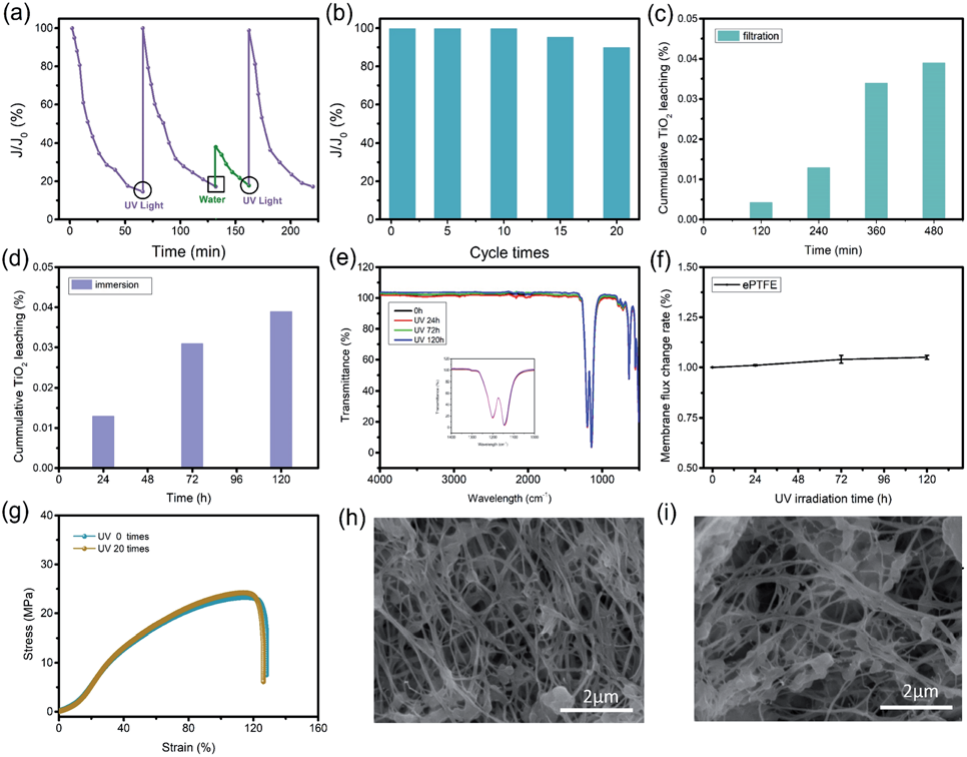
(a) Different treatment methods make the membrane flux change in the filtration process; (b) cyclic test of synergistic filtration and degradation of BSA; leaching experiment of TiO_2_–PAA–ePTFE: (c) filtration and (d) immersion; changes of ePTFE under different UV irradiation (e) FTIR spectrum and (f) pure water membrane flux; (g) stress–strain curve of TiO_2_–PAA–ePTFE before and after UV irradiation; (h) the morphology of TiO_2_–PAA–ePTFE film without UV irradiation after removing titanium dioxide; (i) the morphology of TiO_2_–PAA–ePTFE film after UV irradiation and removal of titanium dioxide.

Ultrafiltration (UF) membranes are generally used for filtering substances with size of 100 to 1000 nm. During the application, most UF membranes will face the problem of channel blockage. Herein, the TiO_2_–PAA–ePTFE films loaded with TiO_2_ particles aim to disclose this problem. The change of membrane flux with time was measured by bovine serum albumin (BSA) to study the clogging and self-cleaning of the TiO_2_–PAA–ePTFE film. BSA will blocking the membrane channels and resulting in the decrease of membrane flux. Then UV light is used to drive the self-cleaning function of the film and restore its flux. The experimental results are shown in Fig. 4a, when continuously filtering 1 g L^−1^ BSA solution, the membrane flux decreased continuously to 20% of its original value after 70 min. At this time, a 365 nm UV light is adopted to the film, and triggering the TiO_2_ to catalyze the BSA decomposition of the blocked film and wash it away. Then, the membrane flux completely recovers to the original level. However, if only pure water is used instead of UV irradiation, the membrane flux can only recover to 40%. And If UV irradiation is re-enabled, the flux can return to the original level after 25 minutes (Fig. S2†). Fig. 4b shows the change of membrane flux with the use times. Surprisingly, the membrane flux can recover to 100% after 10 cycles. Even for 20 times, the self-cleaning function can guarantee ∼90% of membrane flux. The falling off of titanium dioxide will cause secondary pollution of the filtrate, so it is necessary to study the leaching behavior. In the 120 h immersion leaching experiment (Fig. 4c), the leaching rate of titanium dioxide is less than 0.04% (percentage of total titanium dioxide), and in the filtration leaching experiment (Fig. 4d), after the photocatalytic membrane was filtered under UV irradiation for 480 min, the leaching rate of titanium dioxide was less than 0.04%. These results show that the coordination between titanium dioxide and ePTFE is stable and has long-term application prospects. The stability of photocatalytic membrane in ultraviolet light is also one of the very important properties. After 120 h of UV irradiation, the infrared spectrum of ePTFE membranes has seldom change in respected to the original one (Fig. 4e). The intensity of the vibration absorption peaks of CF_2_ in 1046.5 cm^−1^ and 1202.8 cm^−1^ remains constant. The flux change rate of ePTFE membrane is less than 0.1% after 120 h of UV irradiation (Fig. 4f). At the same time, after 20 cycles, the stress–strain curve of TiO_2_–PAA–ePTFE membrane still maintains its original properties (Fig. 4g). These results indicating that ePTFE has excellent UV resistance. The micromorphology of the membrane after UV irradiation has been further observed by SEM. In order to observe the porous structure clearly, the TiO_2_ particles on membrane surface were corroded by concentrated sulfuric acid. As shown in Fig. 4h and i, the membrane used for 20 cycles has the similar microstructure to the freshly prepared one, indicating the PTFE network is stable to the UV irradiation. This photocatalytic induced self-cleaning will provide a new design idea for prolonging the service life of the ultrafiltration membrane.

**Fig. 5:**
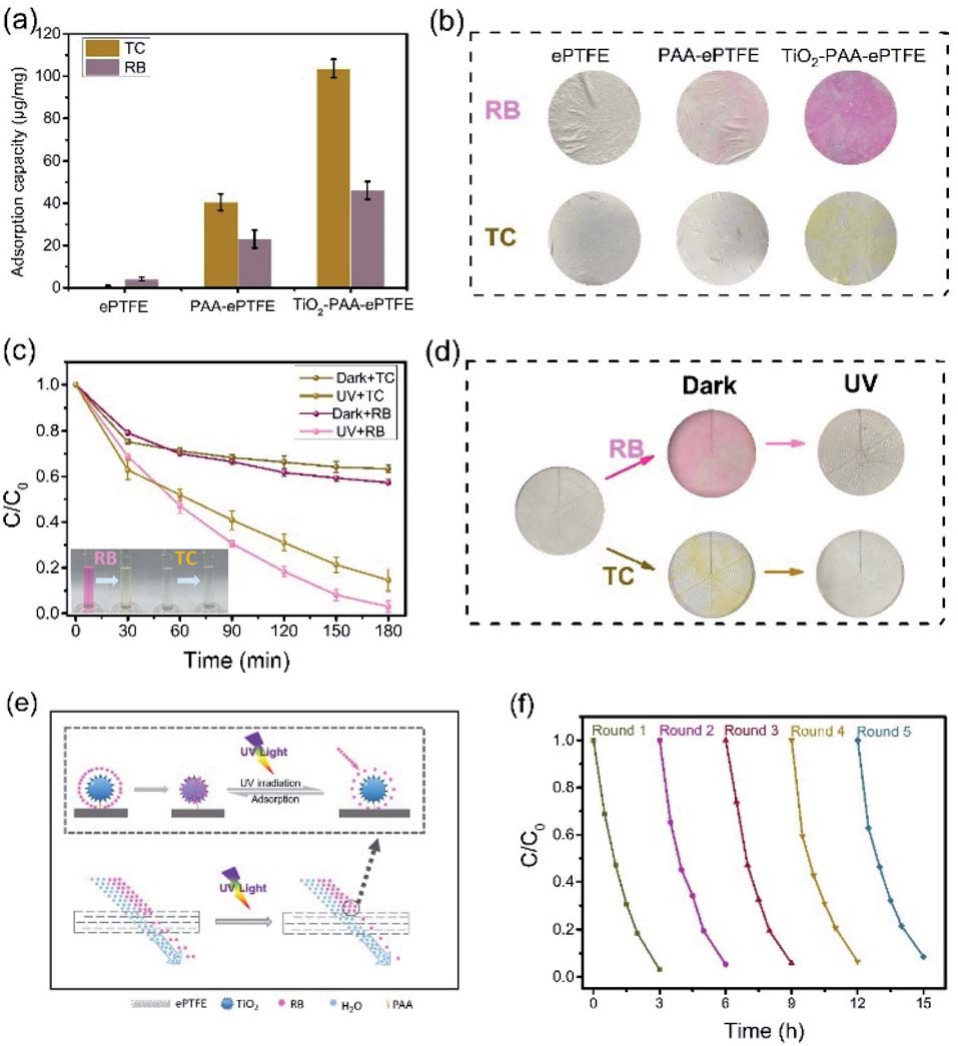
(a) Adsorption rates of three pollutants (RB and TC) adsorbed by different membranes in two hours; (b) adsorption photographs of RB and TC adsorbed by different membranes; (c) synergistic filtration and degradation curves of RB (5 ppm, 20 ml) and TC (15 ppm, 20 ml) under light or dark; (d) photographs of collaborative filtration and degradation of RB and TC in light or dark; (e) schematic diagram of synergistic filtration and catalysis of TiO_2_–PAA–ePTFE for small molecules; (f) cyclic test of synergistic adsorption and degradation of RB (5 ppm, 20 ml).

Generally, filter membrane only retains the substances with size larger than its pore size. But surprisingly, the TiO_2_–PAA–ePTFE film can absorb and decompose small molecular organics. Fig. 5a shows the molecular adsorption capacity of ePTFE, PAA–ePTFE and TiO_2_–PAA–ePTFE films in respect to TC (10 ml, 15 ppm) and RB (10 ml, 5 ppm), respectively. Among them, ePTFE can barely absorb these molecules because of their ultra-low specific surface energy. When modified with PAA and TiO_2_, the adsorption capacity of the modified film to organic molecules is significantly improved. Especially for TiO_2_–PAA–ePTFE film, its adsorption capacity for TC and RB are 103.7 and 46.1 μg mg^−1^, respectively. Fig. 5b shows the real appearance of the ePTFE, PAA–ePTFE and TiO_2_–PAA–ePTFE films after absorbing of RB and TC. The results are consistent with the experimental data, and the color change of TiO_2_–PAA–ePTFE film is the most obvious. Fig. 5c shows the photocatalytic curves of TiO_2_–PAA–ePTFE for RB and TC. For both of them, the residual concentration ratios decrease rapidly in the initial 30 min without UV irradiation. This is due to the adsorption of TiO_2_ to the substrates, which leads to the reduction of the residual in solution. Then, under UV light, photocatalytic effect of TiO_2_ leads to the further reduction of residual concentration ratios to 98% and 83% for RB and TC, respectively. The final treatment volume of RB is 19.9 ml (the initial volume is 20.0 ml). In addition, the antibacterial activity of the TiO_2_–PAA–ePTFE membrane has also been studied. Surprisingly, it was found that its antibacterial activity was as high as 98.2% (Table S1†). As Fig. 5d shows, after UV irradiation, the dyes absorbed on the films have been totally clean out. Thus, the TiO_2_–PAA–ePTFE film has power for degradation of small molecular organics.

Surface adsorption is beneficial to catalytic degradation, while continuous degradation provides power for sustained adsorption. Fig. 5e shows the diagrams of the synergistic effects of filtration and catalysis on small molecular organics. In the filtration process of TiO_2_–PAA–ePTFE membrane, a large number of RB molecules are adsorbed on TiO_2_ due to the adsorption of TiO_2_–PAA–ePTFE, which reduces the RB content in the filtered water. Under UV irradiation, the adsorbed RB decomposes, and TiO_2_ continues to adsorb RB in water, which significantly reduces the RB content in filtered water.

In the application of wastewater filtration, it is very important to evaluate the reusability and stability of the photocatalytic performance of the membrane. As shown in Fig. 5f, after five cycle experiments, it was observed that the degradation rate of RB by TiO_2_–PAA–ePTFE membrane was still about 90%. The film has excellent reusability and stable performance. The reported parameters that relate to recent hybrid membrane development and features of the membranes are summarized in Table 1. The membrane in this work has the outstanding performance in FRR, reusability and pure water permeance.

**Table 1:** Performance comparison between the membranes made in this work and the reported membranes

Membranes	WCA	Feed solution (g L^−1^)	FRR	Pure water permeance (L m^−2^ h^−1^)
PES–TiO_2_@MXene^32^	46°	1 BSA	80.2%	756.8
PVDF–gPAA@FeOOH^33^	20°	1 BSA	93.8%, 4 times	565.8
MCN–PDVF^34^	57°	1 BSA	88%	294.0
PVDF/PBSA^35^	30°	1 BSA	87%	1770.2
PVDF–CND/TiO_2_ (ref. 36)	71°	0.5 BSA	82.6%, 2 times	203.0
PES–Ni–TiO_2_ (ref. 37)	62°	1 BSA	75.4%	871.2
This work	31°	1 BSA	90%, 20 times	1627.0

## Conclusions

In this work, ePTFE film is grafted with PAA chains so that TiO_2_ can immobilized on film surface *in situ*. With the composition of hydrophilic TiO_2_ particles, membrane flux of the obtained TiO_2_–PAA–ePTFE membrane increases significantly from 150 to 1627 L m^−2^ h^−1^ in respect to the pristine ePTFE film. On one hand, the photocatalytic TiO_2_ particles endow the membrane with self-cleaning performance, that is, after surface fouling, the membrane flux can be restored to the original value under UV irradiation. On the other hand, the ePTFE film ensures the stable physical–chemical properties of the membrane. After 20 rounds of UV irradiation, its porous structure still remains intact. Moreover, the hedgehog shaped TiO_2_ particles with high specific surface area are helpful for the membrane to absorb and degrade small organic pollutants such as RB and TC. Overall, the resultant TiO_2_-PAA–ePTFE membrane can be potentially used as a self-cleaning water filtration membrane.

## Author contributions

The manuscript was written through contributions of all authors. All authors have given approval to the final version of the manuscript.

## Conflicts of interest

There are no conflicts to declare.

## Supplementary Material

RA-012-D2RA01026G-s001
