# Supplementary material for: Self-cleaning expanded polytetrafluoroethylene-based hybrid membrane for water filtration
Source: RSC Adv. 2022 May 4;12(21):13228–34. doi: 10.1039/d2ra01026g (PMC9067432; doi:10.1039/d2ra01026g)
Supplement: RA-012-D2RA01026G-s001 [file RA-012-D2RA01026G-s001.pdf]

Electronic Supplementary Material (ESI) for RSC Advances.
This journal is © The Royal Society of Chemistry 2022

Self-cleaning expanded polytetrafluoroethylene-based hybrid membrane for water filtration

Peng Liao [a], Lan You [a], Wen Jiang Zheng *[a], Wei Zou [a], Jie Yan [a], Hu Yang [a], Fan Yang [b]

Supplementary information and experimental methods

Antibacterial experiment

There are 12 groups of samples in the antibacterial experiment. Three groups of standard control, three groups of TiO_2-PAA-ePTFE films were placed in the dark, and the same six groups were placed under UVA. Among them, medical grade polyethylene film (PE) is used as the standard control. Put each sample into a clean plate with the test surface facing upward, accurately measure 0.4ml of inoculation solution with a pipette and drop it onto the surface of each sample, carefully cover it with a film, adjust the film to disperse the bacteria evenly, in which the grafting solution is Enterobacter suspension, and the nutrious Brown prepared is 500 times water dilution (0.2% nutrious Brown). Turn on the black light (UVA) and adjust the power so that the illumination intensity reaches 0.01mW/cm^2 on the surface of the sample. After 24 hours of illumination, 20ml phosphate buffered normal saline eluent was added respectively to fully elute. After diluting the eluent, culture again, and use the colony counter to record the corresponding colony number. The colony count is calculated by colony forming unit (CFU), and its Antibacterial rate (R_a) and Photocatalytic antibacterial contribution rate (R_c) are as follows:

$$R_a=[(C_0-C_1)/C_0]x100 \qquad (1)$$

$$R_c=[(B_1-C_1)/B_1]\ x100 \qquad (2)$$

where C_0 is the value of viable bacteria count (CFU) of control sample after culture under UVA conditions, C_1 is the value of viable bacteria count (CFU) of photocatalytic sample after culture under UVA conditions, and B_1 is the value of viable bacteria count (CFU) of photocatalytic sample after culture under dark conditions.

Membrane characterization

Precision electronic universal material testing machine (Shimadzu AGX-V, JP) was used to analyze the mechanical properties of the film. Synchronous thermal analyzer (Mettler Toledo TGA / DSC 3$^+$, CH) was used to analyze the thermal properties of the films.

Filtering illumination optimization experiment

Put the TiO_2-PAA-ePTFE membrane into a 50ml ultrafiltration cup and continuously filter 1g/L bovine serum albumin. When the membrane flux decreases to 20%, take out the membrane and irradiate it with ultraviolet light (100W, 365nm) for different times, then immerse the membrane in ethanol for ultrasonic cleaning for 1 minute, and finally clean it with ultrapure water. Filter 1g /L bovine serum protein again and measure its membrane flux recovery rate.

Supplementary information tables and figures

Table S1 Antibacterial experiment results of TiO_2-PAA-ePTFE film

	Medical grade PE		TiO_2-PAA-ePTFE	
Light source	Dark	UVA（C_0）	Dark (B_1)	UVA (C_1)
Average colony (CFU)	$8.6x10^8$	$4.8x10^8$	$1.1x10^7$	$8.6x10^5$
R_a		98.2%		
R_c		92.2%		

It can be seen from **Table S1** that the antibacterial rate (R_a) of TiO_2-PAA-ePTFE film under UV is as high as 98.2%, indicating that the film can kill bacteria under UV irradiation. The excellent antibacterial ability of the film making it a wide application prospect of water treatment. At the same time, it can be seen that the antibacterial rate (R_C) contributed by photocatalysis is 92.2%. The loaded titanium dioxide provides most of the antibacterial effect, suggesting that titanium dioxide has high activity under UV. It is consistent with the experimental results of photocatalytic degradation.

Table S2 Mechanical properties and thickness of different films

	Thickness (mm)	Elongation at break (%)	Tensile strength (MPa)	Young's modulus (MPa)
ePTFE	0.033±0.001	145.28±1.25	14.29±0.12	0.029±0.002
PAA-ePTFE	0.034±0.002	144.57±1.61	14.51±0.32	0.039±0.003
TiO_2-PAA-ePTFE	0.039±0.003	119.93±2.12	26.21±0.40	0.17±0.011

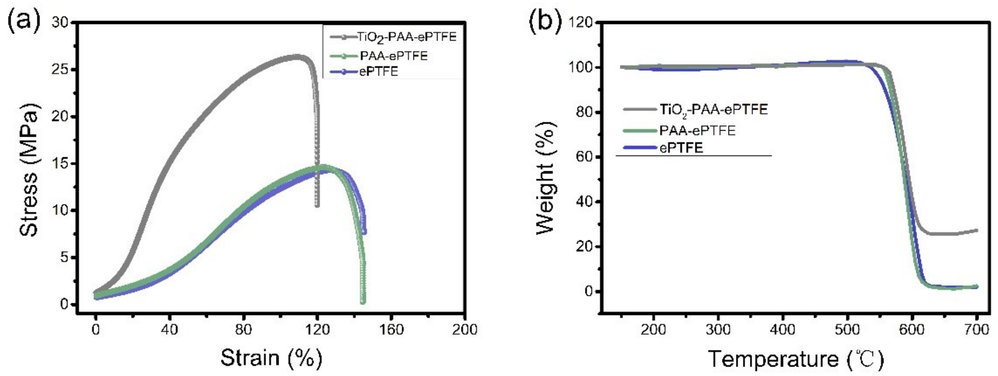

Figure S1. (a)stress-strain curves and (b)thermogravimetric curves of different films

The stress-strain curves of different modified ePTFE are shown in **Figure S1a**， and the tensile strength, elongation at break and Young's modulus are listed in **Table S2**. With the grafting of PAA and $TiO_{2,}$ the tensile strength of ePTFE increases from 14.29 MPa to 14.51MPa and 26.21 MPa, respectively, and the elongation at break decreases. The results show that the addition of titanium dioxide can significantly improve the tensile strength of ePTFE and make the membrane run stably under high pressure. **Figure S1b** shows the thermogravimetric curves of three different films. The thermal stability of modified ePTFE has little change, indicating that the modification will not affect the excellent thermal stability of ePTFE. Among them, the quality of TiO_2-PAA-ePTFE film will not decrease after being reduced to 25%, because of the existence of titanium dioxide.

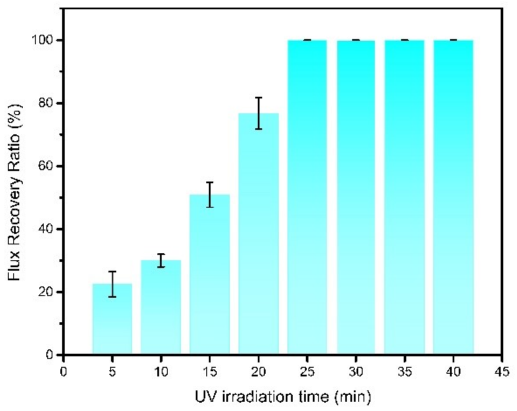

Figure S2. Membrane flux recovery rate of TiO_2-PAA-ePTFE under different UV irradiation time

As shown in **Figure S2,** with the increase of UV irradiation time, the recovery rate of membrane flux gradually increases. When the UV irradiation time reaches 25 min, the membrane flux can be completely recovered, showing excellent self-cleaning ability of the film.
